# Supplementary material for: Alterations in Plasma Lipidomic Profiles in Adult Patients with Schizophrenia and Major Depressive Disorder
Source: Medicina (Kaunas). 2022 Oct 24;58(11):1509. doi: 10.3390/medicina58111509 (PMC9697358; doi:10.3390/medicina58111509)
Supplement: Supplementary file 1 [file medicina-58-01509-s001.zip › Supplementary Figures S1 and S2.pdf]

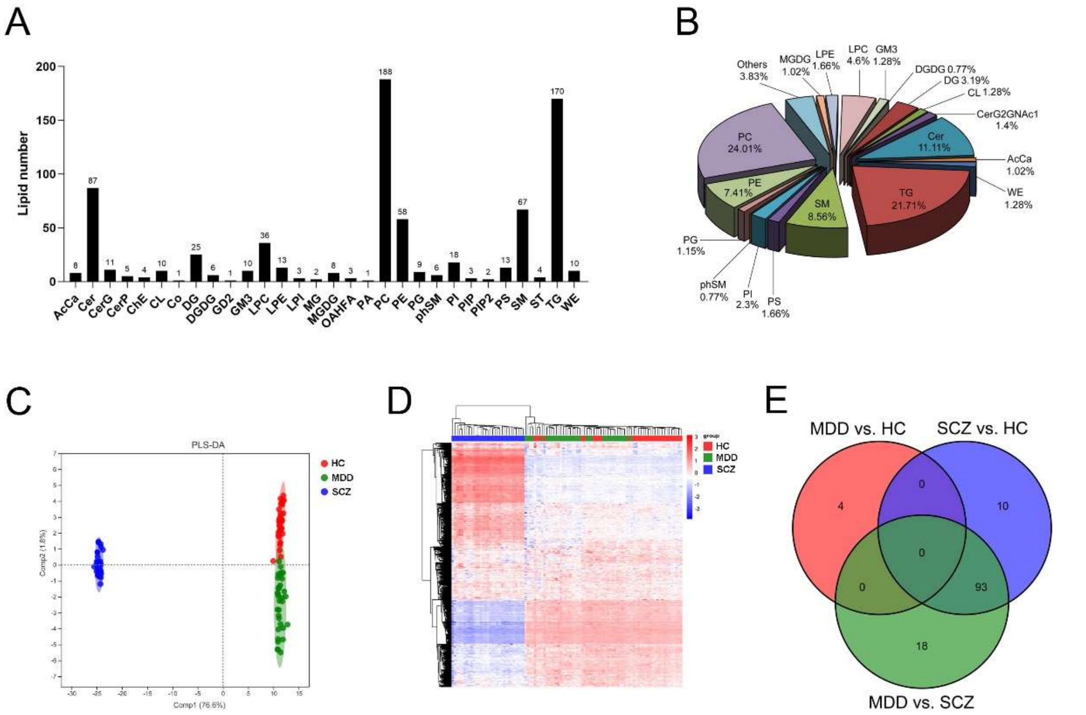

Figure S1. (**A**) Number of identified lipid subgroups and lipid molecules in this experiment and (**B**) percentage composition of lipid content in each lipid class. (**C**) Scatter plot of OPLS-DA model for three groups, (**D**) heatmap analysis for lipid composition in three groups, and (**E**) coverage of lipids in all three comparisons

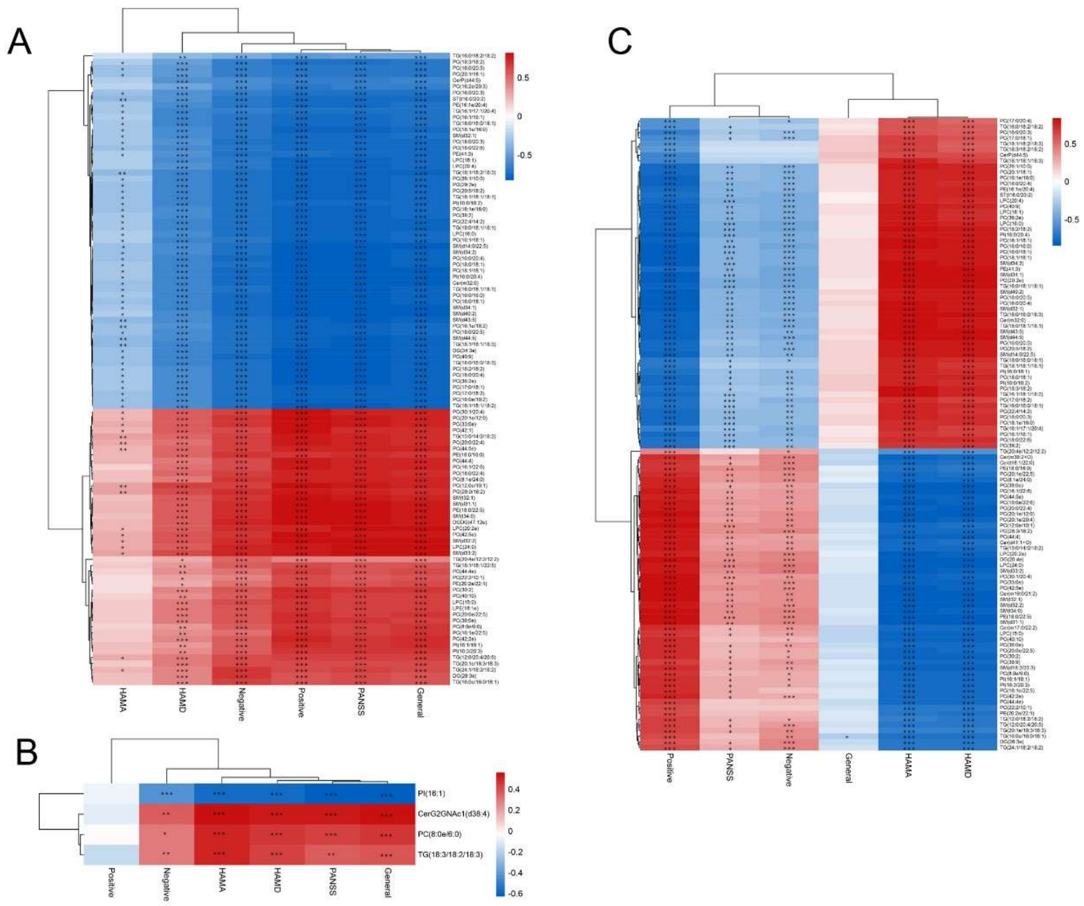

Figure S2. Correlation between clinical parameters and levels of discriminated lipids between SCZ and HC (**A**), MDD and HC (**B**), and SCZ and MDD (**C**).
