# Supplementary material for: Alterations in Plasma Lipidomic Profiles in Adult Patients with Schizophrenia and Major Depressive Disorder
Source: Medicina (Kaunas). 2022 Oct 24;58(11):1509. doi: 10.3390/medicina58111509 (PMC9697358; doi:10.3390/medicina58111509)
Supplement: Supplementary file 1 [file medicina-58-01509-s001.zip › Supplementary Table S2 - Correlation.pdf]

Supplementary Table S2. Correlation between clinical symptoms and levels of lipid classes in the plasma

Lipid	HAMA		HAMD		Positive score		Negative score		General score	
	r value	*P* value	r value	*P* value	r value	*P* value	r value	*P* value	r value	*P* value
OAHFA	-0.133	0.283	-0.089	0.473	0.084	0.513	0.188	0.140	0.101	0.432
WE	-0.067	0.592	0.021	0.866	0.144	0.259	0.018	0.890	0.055	0.668
AcCa	-0.606	0.000	-0.578	0.000	-0.374	0.003	-0.424	0.001	-0.518	0.000
DG	0.074	0.551	0.082	0.511	0.140	0.272	0.223	0.078	0.157	0.220
TG	0.199	0.106	0.102	0.412	0.009	0.945	0.120	0.349	0.078	0.545
MG	0.141	0.255	0.159	0.199	-0.075	0.561	0.127	0.323	0.003	0.984
PC	-0.036	0.771	-0.060	0.630	-0.067	0.602	-0.061	0.632	0.025	0.844
PI	0.159	0.199	0.168	0.173	0.030	0.816	0.016	0.899	0.108	0.401
PE	-0.210	0.087	-0.202	0.102	-0.273	0.031	-0.280	0.026	-0.210	0.099
PS	-0.425	0.000	-0.436	0.000	-0.538	0.000	-0.532	0.000	-0.478	0.000
PG	-0.166	0.180	-0.120	0.334	0.157	0.218	0.082	0.525	0.161	0.208
LPC	0.164	0.185	0.026	0.832	0.293	0.020	0.370	0.003	0.352	0.005
CL	-0.132	0.287	-0.058	0.641	-0.117	0.359	-0.185	0.147	-0.170	0.182
LPE	0.068	0.586	-0.071	0.570	0.343	0.006	0.414	0.001	0.347	0.005
LPI	-0.130	0.293	-0.124	0.318	0.187	0.141	0.208	0.102	0.281	0.026
PA	0.190	0.123	0.167	0.176	0.156	0.223	0.105	0.413	0.233	0.066
PIP	0.338	0.005	0.331	0.006	0.407	0.001	0.345	0.006	0.447	0.000
PIP2	0.232	0.058	0.243	0.047	0.297	0.018	0.172	0.179	0.263	0.037
ChE	0.104	0.404	0.016	0.898	0.039	0.764	0.000	0.998	0.093	0.469
DGDG	0.006	0.963	0.077	0.536	0.291	0.021	0.242	0.056	0.210	0.099
MGDG	-0.165	0.182	-0.210	0.089	-0.337	0.007	-0.325	0.009	-0.225	0.076
Co(Q10)	-0.172	0.165	-0.133	0.285	-0.050	0.699	-0.033	0.795	-0.039	0.764
SM	-0.096	0.441	-0.095	0.443	-0.154	0.227	-0.069	0.591	-0.073	0.569
ST	-0.204	0.097	-0.147	0.234	-0.226	0.075	-0.236	0.063	-0.165	0.197
Cer	-0.152	0.218	-0.212	0.085	0.203	0.111	0.284	0.024	0.186	0.144
CerG	-0.193	0.118	-0.219	0.074	-0.138	0.282	-0.156	0.223	-0.193	0.130
GM3	0.296	0.015	0.325	0.007	0.067	0.603	0.193	0.130	0.147	0.250
phSM	-0.042	0.739	-0.151	0.222	-0.160	0.210	-0.094	0.464	-0.173	0.174
CerP	0.187	0.129	0.108	0.383	0.042	0.744	0.073	0.569	0.147	0.252
GD2	0.194	0.116	0.205	0.096	-0.113	0.378	-0.090	0.485	-0.013	0.917
